# Study on medical professionals’ acceptance of and factors influencing drone delivery for medical supplies

**DOI:** 10.3389/fpubh.2025.1571904

**Published:** 2025-06-03

**Authors:** Zhao Zhang, Chun-Yan Xiao, Ya Wang, Wan-Cui Song, Jia-Yi Sun, Zhi-Guo Zhang

**Affiliations:** ^1^School of Logistics, Chengdu University of Information Technology, Chengdu, China; ^2^Xichang People's Hospital, Xichang, China; ^3^Mianyang Teacher's college, Mianyang, China; ^4^School of Emergency Science, Xihua University, Chengdu, China

**Keywords:** drone delivery, medical supplies, healthcare workers, binary logistic, fuzzy-ISM, management construction pathway

## Abstract

**Introduction:**

Drone delivery technology provides innovative alternatives for the transportation of medical supplies. Due to their quick reaction, effectiveness, and adaptability, drones can drastically reduce the time it takes to deliver medications, vaccines, and emergency supplies as compared to standard logistics models. However, the real marketing of drone delivery technology has been hampered by medical professionals’ concerns about its stability, safety, and dependability. Medical professionals are the primary users of medical delivery drones, hence, their opinions are crucial for the spread of the technology. To encourage the optimization and real-world use of this technology, it is crucial to methodically investigate the elements influencing medical staff’s approval of drone delivery.

**Methods:**

Using a sample of medical personnel from the emergency department of a large hospital in Chengdu City (N=289), this study conducted regression analyses using a binary logistic model for each of the four categories of medical supplies to identify key factors that can influence medical personnel’s willingness to use drones. We then continued to explore the hierarchical structure and dynamic dependency relationships among the deeper factors of these causes using a Fuzzy-ISM model.

**Results:**

The study concluded that, in the initial stage, priority should be given to creating a favorable development environment for the project rather than pursuing substantive construction; in the trial operation period, the focus of construction should be on strengthening infrastructure and improving professional staffing; in the scale-up stage, the focus of attention should be on cost reduction, improving distribution efficiency, improving project safety, and optimizing the way of managing and operating the project.

**Discussion:**

These improvements will be more conducive to increasing the willingness of medical workers to use drones to distribute medical materials and more conducive also to the early realization of regular distribution of medical materials by drones.

## 1 Introduction

The sensitivity of medical institutions to the delivery time of medical supplies is extremely high, especially in public health emergencies, natural disasters, or emergency medical rescue scenarios. The traditional logistics system is difficult to respond to efficiently due to geographic restrictions, transportation obstacles, and insufficient resource allocation (1). In urban areas, the “last-kilometer” distribution is limited by traffic congestion, inefficient distribution, and rising costs, which significantly affect the timely delivery of medical supplies (2). Meanwhile, the issue of lagging medical supplies in remote areas is still prevalent, and high-cost, inefficient distribution further exacerbates the imbalance in the distribution of medical resources (3, 4). Especially during the pandemic and other emergencies, the demand for medical supplies distribution is highly concentrated and unpredictable, which puts forward higher requirements for the existing logistics system (5). However, the technological complexity of cold chain logistics requirements and the safety and integrity that must be ensured during drug distribution make management and implementation much more challenging (6, 7). The lack of synergy between the logistics network and the industrial layout can also constrain the ability to supply medical devices efficiently (8, 9). Taken together, the shortcomings of medical material distribution in terms of efficiency, safety, cost control, and coverage have become an important bottleneck affecting the improvement of medical services. In this context, the development of efficient, flexible, and economical medical supplies distribution solutions has become one of the core problems to be solved by academia and industry.

Equipped with high mobility, rapid deployment, and precise operation capability, Unmanned Aerial Vehicle (UAV) technology has been widely used in agriculture, environmental monitoring, disaster rescue, and other fields (10–13). In agricultural production, UAVs improve operational efficiency and optimize resource utilization through precise spraying and seeding (14); in environmental monitoring and disaster assessment, UAVs’ real-time data acquisition capability provides technical support for rapid response to complex terrain and emergencies (15). In addition, with the deep integration of artificial intelligence and the Internet of Things, the level of intelligence of UAVs continues to improve, and the boundaries of their application in automation tasks continue to be expanded. In the field of logistics, drones break through traditional transportation limitations, providing efficient solutions for the transportation of goods in complex terrain and remote areas. As an innovative tool for “last-mile” delivery, drones meet diversified logistics needs with higher efficiency (16). From commercial courier trials to emergency material transportation, UAVs have demonstrated outstanding flexibility and reliability, and their application scenarios are continuously expanding with the continuous strengthening of technology and policy support, providing a brand new path for the optimization and improvement of the traditional logistics model. According to studies, UAVs are quite beneficial when it comes to distributing medical supplies, and their adaptability and quick reaction times can be extremely helpful in remote locations and during public health situations (17). UAVs can transport vital items precisely by circumventing ground traffic limits; their quick deployment can significantly cut down on delivery time, saving vital time for medical rescue (18).

This technology is still in the experimental stage, though, and there are numerous obstacles to its widespread use in the medical industry. There is a risk of a “feasibility-approval” disconnect in the implementation of this technology because existing research focuses more on optimizing technology performance (e.g., UAV range, path planning, etc.) than it does on examining the technology’s actual effectiveness and feasibility in medical scenarios. Additionally, there is a dearth of systematic research on the acceptance of medical personnel, who are the primary decision-makers in medical scenarios. This study identifies the key factors affecting medical personnel’s willingness to use drone delivery through a mixed-method approach. This study further explores the hierarchical dependencies between the deep factors characterized by these influencing factors, framing a phased construction framework to provide theoretical support and scientific guidance for the promotion of the cause of drone delivery of medical materials, as well as the optimization of related technological solutions in the future.

## 2 Literature review

As an important part of the medical system, the distribution of medical supplies directly affects the treatment effect of patients and the operational efficiency of hospitals (18). An efficient medical material distribution system can ensure the timely supply and rational allocation of resources, alleviate the problem of uneven distribution of medical resources among regions (19), and at the same time, improve the quality of medical services and effectively reduce the waste of resources and operating costs (20). From the existing research, Wang and other scholars believe that optimizing the stockpiling and distribution mechanism of medical supplies can significantly improve the response efficiency of public health events (21), Yani et al. (22) believe that optimizing the supply chain management of medical supplies can effectively reduce the waste of resources and improve the efficiency of the work, and Aggarwal et al. (23) also realized that the distribution of medical materials is directly related to the rational utilization of resources and cost control and that an effective distribution strategy can significantly reduce inventory costs and resource wastage and improve the utilization of materials. In the case of Lucchese and other scholars, the dual reduction of transport and warehousing costs by a hub network was verified in the case of healthcare distribution in Apulia, Italy, by integrating a facility siting algorithm with a path planning model (24).

Despite the growing importance of medical supplies distribution, the existing work system still faces many challenges. Yani et al. (22) believe that if the supply chain is not managed properly, it may lead to delays in surgery, interruptions in patient care, and an increased risk of medical errors. Meanwhile, currently, most hospitals have insufficient cross-sectoral and cross-regional collaboration in medical supplies, which may lead to inefficient resource allocation and reduced healthcare efficiency (25). In their study of the quantity and value of unused medical supplies in the emergency department, Muldoon et al. (26) revealed that there is a large amount of waste of medical supplies used in the emergency department, and that feasible recycling and redistribution strategies must be employed to minimize the waste of the items in question. In general, medical supplies encompass various pharmaceuticals, medical consumables, blood samples, organs, and other biological materials. Storage conditions and inventory levels vary significantly across different types of materials. For instance, traditional pharmaceuticals can be stored in bulk, whereas organ transplants require cold-chain transportation. Therefore, specialized storage facilities and distribution protocols must be designed for distinct medical supplies, which substantially increases the complexity of logistics management (27).

With the continuous development of drone technology, the use of drones for inspection, security, and delivery is becoming more and more common (28). The use of drones for distribution has the advantages of shortening delivery time, reducing costs, and improving flexibility and sustainability (29), and from the existing data, it has a significant effect on improving the efficiency of the “last-kilometer” distribution and customer satisfaction (30). Hospitals are adopting drones to transport medical materials due to their agility and efficiency in overcoming urban traffic limitations, particularly for high-frequency, low-volume on-demand deliveries (31, 32). Additionally, drones demonstrate significant time and efficiency advantages when delivering medical equipment and medications in emergency situations (33). The study by Bhatt et al. (34) confirms the ability of drones to rapidly deliver blood to remote areas, organs, and emergency medical supplies. Furthermore, an empirical study by Nisingizwe et al. (35) for sub-Saharan Africa showed that based on the analysis of data from 12,733 shipments, products were delivered by drones 79–98 min earlier than by road (*p* < 0.001) and that blood and blood product expiry rates were reduced by 67% (95% CI −11.8 to −2.4), moreover demonstrating the value of the technology in geographically complex areas. In contrast, Sumit Aggarwal and Sumit et al. investigated the potential of drone distribution of medical supplies under restricted and controlled environmental conditions and found that the use of drones to transport medical supplies in remote areas could significantly reduce turnaround times for viral load testing. However, the cost of implementing a UAV-based system is significantly higher than traditional transport methods (36). As for the integrity of biological samples transported by drones, controlled experiments at Nagasaki University, Japan, demonstrated that cold ischemia time and key biochemical indicators of rat liver transported by drones were not significantly different from conventional transport, and further studies including large animal experiments may lead to future clinical applications (37).

Preliminary evidence has been obtained for cost-effectiveness analyses of UAVs. For example, Johannessen presented a new conceptual approach to assess the time savings and cost power competition of drones when transporting biological samples; while he argued that drone solutions provide marginal benefits in short-distance transport, the time savings of drones are even more encouraging in a long-distance transport model with proper scheduling and sufficiently high drone speeds (38). Röper, Johann WA, et al. (39) developed formulas for calculating the costs of fixed and aerial delivery AED networks using the example of a rural area in Vorpommern-Greifswald, Germany, and his study showed that the cost of drone-based AED delivery is always lower than that of fixed sites for the same response time. Zailani’s team, on the other hand, compared the cost of transporting blood by drone and ambulance, showing that the cost of drone deployment was higher than that of ambulance in emergencies between a district hospital and the nearest tertiary hospital. However, the costs were offset by significantly reduced travel time as an indicator of the final outcome (40).

Despite the many advantages in terms of efficiency and cost, the scaled application of drone technology in medical scenarios still faces multidimensional challenges. On the technical level, Aggarwal et al. (23) believe that UAVs have limited load capacity, insufficient endurance, and insufficient ability to adapt to complex terrain, and they analyzed the case of UAVs used for the delivery of vaccines and medicines in northeastern India, and concluded that different terrains and types of materials will have an impact on the selection of UAV models and delivery modes. Lim et al. (41) believe that, when UAVs face inclement weather or complex environments, the delivery efficiency will be significantly reduced. Aggarwal and Sumit in their study also found that UAV operations are significantly affected by weather conditions, especially rainfall and high-speed winds (42). In terms of regulation, Rejeb et al. (29) argue that the complexity of laws and regulations governing airspace management and drone use in countries around the world may hinder the widespread use of drones, and Nyaaba et al. (43) argue that drone operations in urban areas may pose a challenge to personal privacy and data protection. In terms of operation, the initial investment and operation cost of drone delivery is high (28), and the related operations are in the experimental or trial operation stage, and there is a lack of mature research on the cost–benefit analysis of this type of activity. Grote et al. (44) also believe that most of the current drone delivery activities are in the experimental stage, and there is a lack of an effective management mechanism for the normalized operation, which needs to be further explored in future work. In the future, reasonable solutions to these problems caused by technology and operations can help the promotion of drones in the field of distribution of medical supplies to a greater extent.

The successful promotion of drone technology not only relies on technological progress and operational maturity but also needs to be understood and accepted by the majority of medical workers. Therefore, it is still very important to conduct in-depth research on the views of medical workers on the distribution of medical supplies by drones. Exploring the adoption behavior of health information systems, Luo et al. (45) found that both perceived ease of use and perceived usefulness significantly influenced medical staff’s willingness to adopt technology, and that hospital size and financial status played a moderating role. Sham’s study showed that most medical personnel have a positive attitude toward drone delivery of general medical supplies, believing that drones can increase the speed of access to supplies in emergency situations (46). While drone technology offers time-saving advantages, other studies have further highlighted medical personnel’s concerns, including reliability gaps, regulatory uncertainties, privacy challenges, and safety risks. Additionally, despite its efficiency, the high operational costs may hinder widespread adoption (43, 47). Some medical personnel have expressed doubts about the stability and temperature-control technology of drone transport when transporting some medical materials with high storage requirements (e.g., vaccines, organs, blood, and other materials that require cold-chain preservation) (46). In Sham’s study, it was also argued that the ‘impersonal’ characteristics may lead patients to question the humane care of medical services (46). This may result in a decrease in the acceptance of medical personnel for drone delivery of medical supplies.

In general, the use of drones to deliver medical supplies can improve the efficiency of medical supply delivery and play a positive role in improving the healthcare supply chain, but some medical workers have a negative evaluation of this work due to current technological and regulatory limitations. The existing literature has identified which elements make medical personnel concerned about the activity of drone delivery of medical supplies. However, to the best of our knowledge, there is still a lack of analysis from a systems perspective of how these elements interact to influence the judgments made by medical personnel. Therefore, in subsequent work, strengthening this aspect of research from a systems analysis perspective could be more effective in enhancing user acceptance on the one hand and maybe more conducive to the wider dissemination of this activity on the other.

## 3 Theoretical foundation

Selecting a suitable theoretical basis is essential to building an analytical framework in order to comprehend medical personnel’s desire to use medical drones to transport medical supplies and the elements influencing it. In order to provide theoretical support for the subsequent factor identification and mechanism analysis, this study builds an explanatory pathway for medical staff’s technology adoption behavior using the Technology Acceptance Model (TAM) and the Diffusion of Innovations (DOI) theory.

### 3.1 Technology acceptance model (TAM)

TAM, proposed by Davis in 1986, is a classic model for studying user behavior when adopting information technology (48). According to the model, user willingness to adopt technology is mainly affected by two core variables: Perceived Usefulness and Perceived Ease of Use. Together, these affect user attitudes and behavioral intentions, ultimately influencing actual use behavior.

In the context of medical drone delivery, Perceived Usefulness can be seen in whether medical personnel believe that drones can improve delivery efficiency, ensure timeliness, and meet urgent medical needs. Perceived Ease of Use reflects their opinions on the ease of operation, learning costs, and compatibility with existing workflows. Perceived Ease of Use reflects the perceived ease of operation, learning costs, and compatibility with existing workflows. It has been pointed out that people often weigh the convenience of their work and risk-taking based on subjective judgment when faced with a new delivery system (49, 50).

In addition, subsequent development versions of the TAM model (e.g., TAM2, UTAUT, etc.) further introduced variables such as social influence, facilitating conditions, and self-efficacy to enhance the explanatory power of the mode (51, 52). In the questionnaire design and data analysis of this study, several variables (e.g., risk perception, policy support, etc.) have a high degree of fit with the core structure of the TAM, which can provide structural support for analyzing the adoption behaviors of medical professionals.

### 3.2 Diffusion of innovation (DOI) theory

The diffusion of innovations (DOIs) theory was proposed by Rogers in 1962 to explain how innovations gradually spread through social systems through specific channels (53). The theory proposes that the process of adoption of new technology by an individual or organization can be divided into five stages: knowledge, persuasion, decision-making, implementation, and confirmation. Their adoption behavior is influenced by five key attributes: Relative Advantage, Compatibility, Complexity, Trialability, and Observability (54).

The theory of diffusion of innovations is highly applicable in the diffusion of medical drone delivery systems. Whether the technology can demonstrate obvious efficiency advantages (relative advantages) over traditional delivery methods is an important factor affecting the adoption of medical staff; at the same time, its degree of compatibility with the hospital’s existing management processes and technology systems directly affects the difficulty of the actual promotion; furthermore, the complexity of the system’s operation (complexity) determines the medical staff’s learning and adaptation costs; and the establishment of a pilot project provides an opportunity for experimentation and observation. The establishment of a pilot project provides experimentation and observability and provides a reference point for those who have not yet adopted it. This theory also provides theoretical support for the construction of a stage-by-stage evolution model in this study, which clarifies the dynamic path from introduction to acceptance of UAS.

### 3.3 Theoretical integration and applicability to this study

Although TAM and DOI have different theoretical starting points, they are highly complementary in explaining the logical paths of individuals’ behavior toward technology adoption. TAM emphasizes the formation mechanisms of subjective perception and behavioral intentions, while DOI focuses on the external push paths of innovation attributes and organisational diffusion. By combining the two, the logic of medical personnel’s acceptance of the medical drone delivery system can be more comprehensively portrayed, taking into account their subjective judgment at the individual level, as well as incorporating the institutional environment and organisational diffusion mechanism.

Based on the above theoretical support, this paper further introduces the stage evolution theory as a methodological framework and divides the adoption process of the medical drone delivery project into three stages, namely, the “environment construction period,” the “technology optimization period,” and the “operation enhancement period,” which correspond to the three stages of “environment construction period,” “technology optimization period,” and “operation enhancement period” (55–57). The three phases correspond to the different stages of technology diffusion in DOI and the trajectory of perception and willingness to use in TAM, respectively. Through a combination of theory-driven and empirical analyses, this paper seeks to reveal the dynamic evolution of medical personnel’s willingness to adopt and its key influencing factors from a mechanistic perspective.

## 4 Method

### 4.1 Research steps

Existing studies have shown that some medical personnel have concerns about delivering medical items by drone. To investigate the causes behind this, this study will focus on the willingness of medical personnel to use drones to deliver medical items and adopt a quantitative research design that combines binary logistic regression and fuzzy interpreted structural modeling (Fuzzy-ISM). Based on the literature analysis and research interviews, questionnaires were designed and administered to the target group to collect valid data. After identifying the key factors affecting the willingness to use binary logic, Fuzzy-ISM was further used to systematically identify the reasons why medical personnel have concerns about the delivery of medical supplies by drones, and finally, a set of improvement plans was designed at the theoretical level by analyzing the logical relationships between these reasons.

### 4.2 Analysis of surface causes

The reasons for the different attitudes of medical personnel toward the use of drones for the delivery of medical supplies are varied. However, their behavioral decisions ultimately manifest as two distinct endpoints: “willingness to use (1)” or “unwillingness to use (0).” Specifically, the medical personnel’s assessment of the outcome of evaluating multiple influencing factors will directly determine the binary tendency of their willingness to use, which is a typical {0,1} binary decision problem (58). For binary decision-making problems, methods such as Markov chains, algebraic models, or ordered binary decision-making can usually be used for analysis (59). The use of binary logistic regression in this study is mainly based on the following considerations: first, logistic regression does not require pre-set data distribution characteristics, which can better capture the nonlinear effects of variables on the binary results, which is highly consistent with the exploratory research needs of this paper; second, considering the questionnaire design and sample size of the subsequent study, if a very complex method is used for the analysis, the problem of overfitting may occur and reduce the computational efficiency; third, after obtaining a reasonable amount of data, regression analyses can be carried out using binary logistic models (60), which can quantify more intuitively the effects of various reasons on the attitudes of medical personnel toward use, and thus provide data support for subsequent scientific decision-making.

#### 4.2.1 Binary logistic model construction

According to the characteristics of the binary logistic model (61), when analyzing and studying the current willingness of medical workers to use drones to deliver medical consumables, the indicator of the willingness of medical workers to use drones to deliver was set as the dependent variable with the score S. From preliminary team research and existing studies (43, 46, 47), medical workers may have different willingness and views due to the different value and scarcity of medical materials, and the analysis results obtained by categorizing all medical items into one class for research may not be accurate. Therefore, this study will analyze the medical materials independently by dividing them into four classes according to value and scarcity, which are specifically categorized as follows:

1. Low-value non-rare medical materials (e.g., common drugs, serum samples, common vaccines, etc., No. S_1_)
2. Low-value scarce medical materials (e.g., snake venom serums, epidemic prevention materials during the period of epidemic prevention, etc., No. S_2_)
3. High-value non-rare medical materials (e.g., special drugs for some common diseases, special drugs for anticancer, etc., No. S_3_).
4. High-value scarce medical materials (e.g., organs, special drugs for rare diseases, etc., No. S_4_).

When the value of the dependent variable is 1, it means that medical workers are willing to use drones, and when it takes the value of 0, it means that they are not willing to use drones, which leads to the binary logistic regression equation of this paper:


(1) $$LogitP=\ln\left(P/1-P\right)=\beta_{0}+\beta_{1}A_{1}+\beta_{2}A_{2}+\ldots +\beta_{n}A_{n}+\varepsilon$$

P in Equation 1 represents the likelihood that the dependent variable value will equal 1. β_0_ represents the regression coefficient of the independent variable; if β_n_ is positive, it indicates that the nth factor influences willingness positively; if β_n_ is negative, it indicates that the nth factor influences willingness negatively. With *ε* representing the random error, a represents the nth independent variable that influences willingness. Using the dependent variable’s characteristics and the equation that was created, the following fundamental hypotheses are put forth for the model:

*H1*: The willingness of different medical personnel to use drones to deliver medical materials varies. Individual characteristics (e.g., gender, age, etc.), the frequency of urgently needed medical items in their work, and the level of construction of hospitals may affect their willingness to use drones.

*H2*: Medical personnel demonstrate different willingness to use drones when delivering medical items of different value and scarcity.

Consequently, the survey variables and questionnaire design content of this study were established as indicated in Table 1 based on the earlier analysis and exploratory research.

**Table 1:** Design of variables in the survey of medical personnel’s willingness to use drones to deliver medical supplies/questionnaire design table.

No.	Variable	Variable definition	Variable type	Remarks
V_1_	Gender	0 = Female, 1 = Male	Nominal Variable	Personal Characteristics
V_2_	Age	1 = 20–35, 2 = 36–50, 3 = 51–65, 4 = 66+	Ordered Variable	
V_3_	How often do you have a sudden and urgent need for medical supplies at your current job?	1 = Little, 2 = Generally, 3 = Many	Ordered Variable	
V_4_	Does your hospital have/experiment with drone delivery?	0 = No, 1 = Yes	Nominal Variable	Characteristics of the construction of the hospital where they work
V_5_	How often does your hospital urgently dispatch medical supplies from other places?	1 = less, 2 = average, 3 = more	Ordered Variable	
V_6_	How fast is your hospital currently dispatching medical supplies urgently?	1 = slow, 2 = average, 3 = fast	Ordered Variable	
V_7_	Whether your hospital emphasize technological innovation?	0 = No, 1 = Yes	Nominal Variable	
V_8_	What is the efficiency of your current hospital logistics department?	1 = low, 2 = average, 3 = high	Ordered Variable	
V_9_	Whether drone noise affects your work?	0 = No, 1 = Yes	Nominal Variable	Respondents’ perceptions of the use of drones to deliver medical supplies
V_10_	What do you think about the cost of drone delivery?	1 = low, 2 = average, 3 = high	Ordered Variable	
V_11_	Whether you think it is safe to deliver medicines by drone?	0 = No, 1 = Yes	Nominal Variable	
V_12_	Do you trust drones to deliver on time?	0 = No, 1 = Yes	Nominal Variable	
V_13_	Do you trust drones to deliver supplies to exact locations?	0 = No, 1 = Yes	Nominal Variable	
V_14_	Whether there is more policy support for drone delivery in your region?	1 = No, 2 = Unknown, 3 = Yes	Nominal Variable	
V_15_	Do you think the current drone delivery technology is mature?	0 = No, 1 = Yes	Nominal Variable	
V_16_	Are you concerned about the ethical risks associated with drone delivery of medical supplies?	0 = No, 1 = Yes	Nominal Variable	
V_17_	What do you emphasize the most when you need to dispatch medical supplies urgently?	0 = speed, 1 = safety	Nominal Variable	
V_18_	Whether drone delivery of medical supplies will be popularized in the short term?	0 = No, 1 = Yes	Nominal Variable	
V_19_	What kind of situation is more suitable for drone delivery of medical supplies?	0 = Ordinary situation, 1 = Emergency situation	Nominal Variable	
V_20_	Whether you are familiar with or have operated a drone?	0 = No, 1 = Yes	Nominal Variable	
V_21_	Have you heard reports of successful drone delivery of supplies?	0 = No, 1 = Yes	Nominal Variable	
S_1_	Would you be willing to use drones to deliver low-value non-rare medical supplies?	0 = No, 1 = Yes	Nominal Variable	Dependent Variables
S_2_	Would you be willing to use drones to deliver low-value rare medical supplies?	0 = No, 1 = Yes	Nominal Variable	
S_3_	Would you be willing to use a drone to deliver high-value, non-rare medical supplies?	0 = No, 1 = Yes	Nominal Variable	
S_4_	Would you be willing to use drones to deliver high-value, scarce medical supplies?	0 = No, 1 = Yes	Nominal Variable	

#### 4.2.2 Data sources

At present, some large cities in China have already carried out experiments on the distribution of medical supplies by drones, and some of these experiments have already entered the trial operation stage. From the viewpoint of the supporting conditions required for the distribution of medical supplies by drones in the city, the large cities with a higher degree of development and larger populations may have a better chance of regularizing the distribution of supplies in this way earlier (50). Considering that Chengdu City, Sichuan Province, has taken the low-altitude economy industry as a leading development industry in the past 5 years, has recently constructed a civil aviation medical center, and that several hospitals have conducted experiments on the delivery of medical supplies by drones, medical personnel in large hospitals in Chengdu City were selected as the research subjects in this study.

From the relevant literature (62–65) and the preliminary research, the current need to deploy medical supplies from outside the hospital are mostly from emergency department medical personnel, and questionnaires were therefore distributed to medical personnel in the emergency department of several hospitals in Chengdu City, and the effectively recovered data were used as the tabular data for this study in the analysis of the causes. This survey is based on the experience of traditional binary logic to determine the sample size, after determining the sample size (sample size in the number of independent variables about 10 times) (23) in order to obtain the number of dependent variables more than 10 times the number of valid questionnaires while ensuring that the recovery rate is more than 80%: the distribution of questionnaires to the test area was 300, the recovery of valid questionnaires totaled 289 (validity rate of 96.33%), and this was used to analyze the willingness of medical personnel to use drones to carry out medical material distribution..

### 4.3 Analysis of deeper factors

To delve deeper into the logical relationships that exist between the independent variables, and thus to better formulate high-quality strategies, it is necessary to analyze the independent variables must be examined more thoroughly, taking into account the deeper factors that are equally present in each of the variables as well as any potential logical relationships that may exist between these factors as a whole. Through binary logistic analysis, we can intuitively identify which key independent variables significantly impact the dependent variable. However, this model cannot reveal potential interactions between these variables. The Fuzzy-ISM model’s hierarchical deconstruction capability is more appropriate for the development of multi-stage medical technology promotion strategies than DEMATEL’s single quantification of causal strength (66, 67). Additionally, its Delphi-Fuzzy integration mechanism can effectively capture experts’ perceptions of the “moderate influence” and other gray areas of cognitive differences more effectively than ANP’s rigid reliance on weight assignment. Finally, compared to SEM’s large-sample data requirement (68), fuzzy-ISM is still able to extract robust structural insights from small-scale expert data, which precisely matches the status quo of less data in the field of medical UAV delivery. In order to further analyze the interaction between deep elements, this study will employ the fuzzy-ISM approach (69, 70).

#### 4.3.1 Fuzzy-ISM model construction

**Figure 1:**
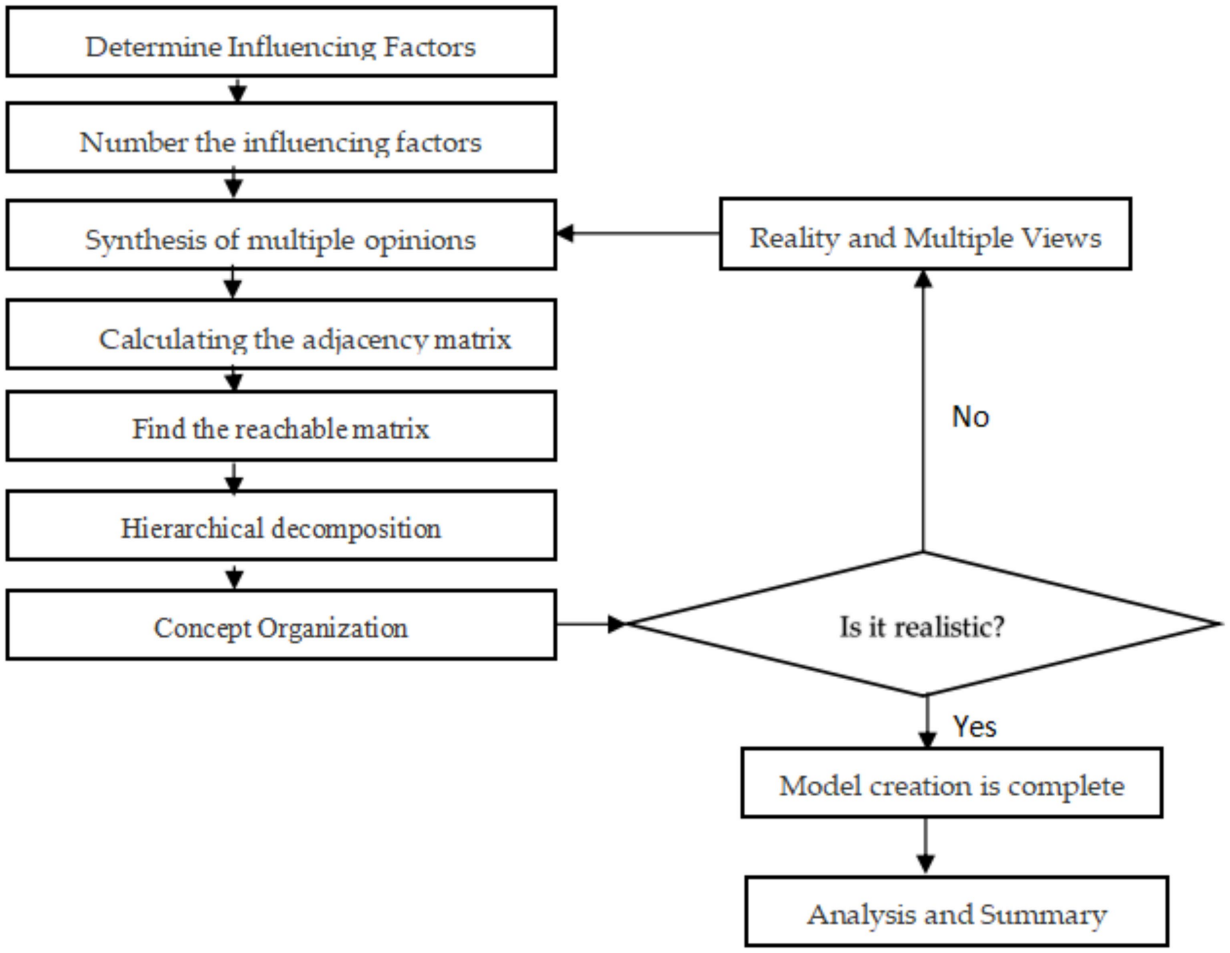
Fuzzy ISM general procedure.

In essence, the traditional ISM is a structural modeling technique that uses the conceptual system of scientific topological operations to represent the mathematical logic of the object of study. This results in a streamlined and highly hierarchical directed topological map that allows analysts to assess the order of the problem of dealing with the object of study and the overall focus of the work and seek an optimal solution to the problem from a global perspective through the final topological map. However, it may contain errors due to the competent judgment of the modeler (71, 72). To address this shortcoming, this study will use the Delphi method to synthesize the research and judgment of the influence conditions, forming the Fuzzy-ISM approach. The general procedure steps of this method are depicted in Figure 1, which aims to minimize the results of personal subjective judgment errors caused by mistakes.

After the process of data collection, processing, adjacency matrix, and reachability matrix solving, the final ISM is formed, at which point the final logical relationship that exists between the factors has been obtained and will be the further basis for the results of this study.

#### 4.3.2 Data sources

After identifying the deep factors, we distributed an expert consultation survey to 35 professors specializing in logistics and healthcare research from universities in Chengdu. The survey aimed to assess interrelationships between these factors. Each participant received detailed guidelines explaining the factors’ definitions and survey completion requirements. All responses were successfully collected. Following standardized ISM procedures, we analyzed the data and derived the final interpretive structural model.

## 5 Results

### 5.1 Binary logistic analysis

#### 5.1.1 Data characterization

A summary of the effectively recovered data from this study is shown in Table 2. From the preliminary survey data, there are large differences in the willingness of medical personnel to use drone delivery when facing four different types of medical supplies. Specifically, the percentage of those willing to use drone distribution when facing low-value non-rare medical supplies is 74.05%, while the percentage of those willing to use drone distribution when facing low-value scarce medical supplies is 33.22%. For high-value items, the agreement rate decreased further. 46.71% agreed with the use of drones to deliver high-value non-rare medical supplies, while only 12.80% supported the use of drones to deliver high-value scarce supplies. The Technology Acceptance Model (TAM) indicated that the perceived usefulness (PU) and perceived ease of use (PEOU) of drone delivery by medical professionals directly influenced their willingness to adopt the technology. For high-value, scarce supplies, medical personnel’s concerns about the safety and reliability of drones significantly weakened their PU. Meanwhile, based on the DOI theory, the relative advantages of drone delivery (e.g., improved timeliness) were limited in complex scenarios, and their enhanced perception of operational complexity (e.g., airspace coordination, emergency response, and other requirements) further reduced the likelihood of technology adoption. This phenomenon is consistent with the findings of established studies (36, 37). To further explore these decision-making patterns, subsequent in-depth analyses of medical personnel’s willingness to deliver medical supplies for each of the four categories will be done separately.

**Table 2:** Statistical results of the preliminary data of the questionnaire.

Variable number	Variable definition	Count	Percentage
V_1_	0	143	0.4948
	1	146	0.5052
V_2_	1	118	0.4083
	2	97	0.3356
	3	62	0.2145
	4	12	0.0415
V_3_	1	140	0.4844
	2	135	0.4671
	3	14	0.0484
V_4_	0	250	0.8651
	1	39	0.1349
V_5_	1	148	0.5121
	2	90	0.3114
	3	51	0.1765
V_6_	1	72	0.2491
	2	162	0.5606
	3	55	0.1903
V_7_	0	92	0.3183
	1	197	0.6817
V_8_	1	117	0.4048
	2	118	0.4083
	3	54	0.1869
V_9_	0	23	0.0796
	1	266	0.9204
V_10_	1	40	0.1384
	2	62	0.2145
	3	187	0.6471
V_11_	0	58	0.2007
	1	231	0.7993
V_12_	0	29	0.1003
	1	260	0.8997
V_13_	0	60	0.2076
	1	229	0.7924
V_14_	1	69	0.2388
	2	149	0.5156
	3	71	0.2457
V_15_	0	97	0.3356
	1	192	0.6644
V_16_	0	229	0.7924
	1	60	0.2076
V_17_	0	116	0.4014
	1	173	0.5986
V_18_	0	225	0.7785
	1	64	0.2215
V_19_	0	41	0.1419
	1	248	0.8581
V_20_	0	87	0.3010
	1	202	0.6990
V_21_	0	60	0.2076
	1	229	0.7924
S_1_	0	75	0.2595
	1	214	0.7405
S_2_	0	193	0.6678
	1	96	0.3322
S_3_	0	154	0.5329
	1	135	0.4671
S_4_	0	252	0.8720
	1	37	0.1280

#### 5.1.2 Conclusion of regression analysis of dependent variables

This study employed the Enter method in SPSS software, constructing four independent binary logistic regression models to evaluate determinants of S_1_-S_4,_ respectively, with key statistical outputs presented in Tables 3–6.

**Table 3:** S_1_ regression results.

Variable number	B	S.E.	Wald	df	Sig.	Exp (B)
V_1_	0.598	0.342	3.063	1	0.080	1.819
V_2_	0.056	0.195	0.081	1	0.776	1.057
V_3_	0.101	0.290	0.121	1	0.728	1.106
V_4_	2.348	0.851	7.610	1	0.006	10.463
V_5_	0.334	0.224	2.208	1	0.137	1.396
V_6_	0.569	0.284	4.004	1	0.045	1.767
V_7_	0.268	0.360	0.555	1	0.456	1.307
V_8_	0.507	0.273	3.448	1	0.063	1.660
V_9_	0.582	0.584	0.994	1	0.319	1.790
V_10_	0.452	0.226	4.017	1	0.045	1.572
V_11_	1.411	0.453	9.689	1	0.002	4.100
V_12_	−0.106	0.544	0.038	1	0.845	0.899
V_13_	0.359	0.393	0.834	1	0.361	1.432
V_14_	0.563	0.253	4.939	1	0.026	1.756
V_15_	0.647	0.415	2.430	1	0.119	1.910
V_16_	0.495	0.435	1.298	1	0.255	1.641
V_17_	1.198	0.336	12.740	1	0.000	3.315
V_18_	−0.022	0.403	0.003	1	0.956	0.978
V_19_	0.165	0.486	0.116	1	0.734	1.180
V_20_	0.690	0.416	2.752	1	0.097	1.993
V_21_	0.171	0.464	0.135	1	0.713	1.186
C	−8.220	1.812	20.583	1	0.000	0.000

B, Unstandardized Coefficients; S.E., Standard Error; Wald, Wald Chi-square; df, Degrees of Freedom; Sig., Significance (*p*-value); Exp (B), Odds Ratio.

**Table 4:** S_2_ regression results.

Variable number	B	S.E.	Wald	df	Sig.	Exp (B)
V_1_	0.564	0.313	3.259	1	0.071	1.758
V_2_	0.185	0.173	1.151	1	0.283	1.203
V_3_	0.660	0.269	6.005	1	0.014	1.935
V_4_	1.125	0.428	6.896	1	0.009	3.080
V_5_	0.857	0.210	16.687	1	0.000	2.356
V_6_	0.216	0.278	0.604	1	0.437	1.241
V_7_	0.373	0.347	1.152	1	0.283	1.452
V_8_	0.469	0.242	3.777	1	0.052	1.599
V_9_	0.696	0.566	1.510	1	0.219	2.006
V_10_	0.480	0.223	4.632	1	0.031	1.616
V_11_	1.385	0.538	6.627	1	0.010	3.993
V_12_	0.526	0.541	0.943	1	0.332	1.691
V_13_	0.295	0.392	0.564	1	0.453	1.343
V_14_	0.515	0.223	5.346	1	0.021	1.674
V_15_	0.976	0.420	5.393	1	0.020	2.654
V_16_	0.017	0.370	0.002	1	0.964	1.017
V_17_	0.523	0.314	2.771	1	0.096	1.687
V_18_	−0.236	0.373	0.402	1	0.526	0.790
V_19_	0.817	0.486	2.826	1	0.093	2.264
V_20_	0.660	0.389	2.871	1	0.090	1.934
V_21_	0.807	0.468	2.978	1	0.084	2.242
C	−13.169	1.962	45.052	1	0.000	0.000

B, Unstandardized Coefficients; S.E., Standard Error; Wald, Wald Chi-square; df, Degrees of Freedom; Sig., Significance (*p*-value); Exp (B), Odds Ratio.

**Table 5:** S_3_ regression results.

Variable number	B	S.E.	Wald	df	Sig.	Exp (B)
V_1_	0.361	0.309	1.367	1	0.242	1.435
V_2_	0.172	0.172	1.004	1	0.316	1.188
V_3_	0.694	0.265	6.841	1	0.009	2.002
V_4_	2.118	0.525	16.287	1	0.000	8.314
V_5_	0.695	0.211	10.822	1	0.001	2.003
V_6_	0.597	0.276	4.668	1	0.031	1.816
V_7_	0.380	0.337	1.271	1	0.260	1.462
V_8_	0.428	0.237	3.268	1	0.071	1.535
V_9_	0.794	0.539	2.172	1	0.141	2.213
V_10_	0.391	0.215	3.324	1	0.068	1.479
V_11_	1.456	0.504	8.352	1	0.004	4.289
V_12_	0.749	0.528	2.013	1	0.156	2.114
V_13_	0.281	0.368	0.585	1	0.444	1.325
V_14_	0.550	0.221	6.191	1	0.013	1.733
V_15_	1.110	0.405	7.528	1	0.006	3.035
V_16_	−0.101	0.370	0.074	1	0.786	0.904
V_17_	0.713	0.308	5.362	1	0.021	2.041
V_18_	−0.858	0.369	5.407	1	0.020	0.424
V_19_	0.853	0.449	3.620	1	0.057	2.348
V_20_	0.553	0.368	2.259	1	0.133	1.739
V_21_	0.756	0.431	3.069	1	0.080	2.129
C	−12.966	1.928	45.241	1	0.000	0.000

B, Unstandardized Coefficients; S.E., Standard Error; Wald, Wald Chi-square; df, Degrees of Freedom; Sig., Significance (*p*-value); Exp (B), Odds Ratio.

**Table 6:** S_4_ regression results.

Variable number	B	S.E.	Wald	df	Sig.	Exp (B)
V_1_	−0.099	0.445	0.049	1	0.824	0.906
V_2_	−0.147	0.249	0.347	1	0.556	0.863
V_3_	0.585	0.397	2.173	1	0.140	1.794
V_4_	1.512	0.538	7.899	1	0.005	4.537
V_5_	0.939	0.287	10.718	1	0.001	2.557
V_6_	0.412	0.414	0.990	1	0.320	1.510
V_7_	0.635	0.529	1.441	1	0.230	1.888
V_8_	0.803	0.362	4.925	1	0.026	2.233
V_9_	0.887	0.939	0.892	1	0.345	2.427
V_10_	0.842	0.366	5.284	1	0.022	2.321
V_11_	2.489	0.995	6.255	1	0.012	12.044
V_12_	−0.081	0.729	0.012	1	0.912	0.923
V_13_	0.653	0.686	0.907	1	0.341	1.921
V_14_	0.835	0.338	6.122	1	0.013	2.306
V_15_	1.246	0.649	3.691	1	0.055	3.477
V_16_	−0.477	0.572	0.694	1	0.405	0.621
V_17_	0.649	0.462	1.970	1	0.160	1.914
V_18_	−0.683	0.607	1.267	1	0.260	0.505
V_19_	0.720	0.754	0.913	1	0.339	2.055
V_20_	1.277	0.651	3.845	1	0.050	3.585
V_21_	0.523	0.770	0.461	1	0.497	1.687
C	−18.085	3.260	30.781	1	0.000	0.000

B, Unstandardized Coefficients; S.E., Standard Error; Wald, Wald Chi-square; df, Degrees of Freedom; Sig., Significance (*p*-value); Exp (B), Odds Ratio.

Where Table 3 corresponds to the S_1_ regression model, the Hosmer-Lemeshaw significance coefficient is 0.956 (greater than 0.05), the model chi-square value is 2.609, Omnibus significance is less than 0.05, the model Cox-Snell R-square value is 0.263, the Negarko Koko R-square is 0.385, and the value of the −2 log likelihood is 242.807.

The regression model in Table 4 corresponding to S_2_ has a Hosmer-Lemeshaw significance coefficient of 0.057 (greater than 0.05), a model chi-square value of 15.136, an Omnibus significance of less than 0.05, a modeled Cox-Snell R-square value of 0.259, a Negolkolko R-square of 0.359, and a − 2 log likelihood value of 280.984.

Table 5 corresponds to the regression model for S_3_ with a Hosmer-Lemeshaw significance coefficient of 0.473 (greater than 0.05), a model chi-square value of 7.609, an Omnibus significance of less than 0.05, a modeled Cox-Snell R-square value of 0.322, a Negolkolko R-square of 0.430, and a − 2 log likelihood value of 287.062.

Table 6 corresponds to the regression model for S_4_ with a Hosmer-Lemeshaw significance coefficient of 0.166 (greater than 0.05), where the model cardinality value is 11.690, the Omnibus significance is less than 0.05, the model Cox-Snell R-squared value is 0.211, the Negolkolko R-squared value is 0.394, and the −2 log likelihood value is 152.809.

From the results of the analysis, the four models were in a good state of fit and can be used for subsequent analysis.

The regression analysis in Table 3 demonstrates that six independent variables significantly influenced S_1_ (Sig. < 0.05). These variables include the following: V_4_ (does your hospital have/experiment with drone delivery), V_6_ (how fast your hospital is currently dispatching medical supplies urgently), V_10_ (what do you think about the cost of drone delivery), V_11_ (whether you think it is safe to deliver medicines by drone), V_14_ (whether there is more policy support for drone delivery in your region), and V_17_ (what do you emphasize the most when you need to dispatch medical supplies urgently).

Interpreted from the TAM and DOI perspectives, the availability of pilot experience demonstrates “Trialability,” whereby hospitals have pilot experiences that make medical staff more familiar with drone operations, thereby increasing perceived ease of use (PEOU). The speed of current scheduling is closely related to the relative advantage of drones. When traditional scheduling is inefficient, drones have a clear advantage in terms of timeliness, which improves their perceived usefulness (PU). Cost reduction and safety enhancement positively influence decision-making by optimizing technology utility perceptions. Policy support, in turn, builds system trust through enhanced technology-environment compatibility.

From the regression results in Table 4, the independent variables with Sig. values less than 0.05 had a significant effect on S_2._ These independent variables include: V_3_ (how often do you have sudden and urgent need for medical supplies at your current job), V_4_ (does your hospital have/experiment with drone delivery), V_5_ (how often does your hospital urgently dispatch medical supplies from other places), V_10_ (what do you think about the cost of drone delivery), V_11_ (whether you think it is safe to deliver medicines by drone), V_14_(whether there is more policy support for drone delivery in your region), and V_15_ (do you think the current drone delivery technology is mature).

In particular, the frequency of demand for emergency supplies at work was positively associated with the degree of performance improvement in drone delivery. The higher the frequency of demand, the more medical staff perceive the advantage of improved delivery efficiency by drones, the greater the perceived usefulness (PU). Hospital pilot experience, cost, safety, and policy factors were consistent with the explanations in the S_1_ model and still corresponded to the concepts of testability, relative advantage, complexity, and compatibility. Overall, the perceived advantage of drone delivery increases in contexts of high demand frequency and technological maturity, driving adoption; the opposite weakens its relative advantage.

From the regression results in Table 5, the independent variables with Sig. values less than 0.05 had a significant effect on S_3._ These independent variables include the following: V_3_ (how often do you have sudden and urgent need for medical supplies at your current job), V_4_ (does your hospital have/experiment with drone delivery), V_5_ (how often does your hospital urgently dispatch medical supplies from other places), V_6_ (how fast your hospital is currently dispatching medical supplies urgently), V_11_ (whether you think it is safe to deliver medicines by drone), V_14_ (whether there is more policy support for drone delivery in your region), V_15_ (do you think the current drone delivery technology is mature), V_17_ (what do you emphasize the most when you need to dispatch medical supplies urgently), and V_18_ (whether drone delivery of medical supplies will be popularized in the short term).

The impact of these short-term penetration expectations is consistent with the Observability property of the DOI theory. When medical professionals expect rapid diffusion of drone delivery, it indicates that they have observed or foreseen the potential benefits of the technology, which can positively influence their adoption intentions. The mechanisms of the remaining factors are consistent with the previous findings: high frequency of demand drives adoption by reinforcing perceived usefulness, while established pilot experience lowers the technological threshold by improving ease of use. The balance between dispatch efficiency and cost security reflects the relative advantages of drones and the complexity of implementation, and policy support continues to reflect the compatibility of the technology solution with the institutional environment. Additionally, the tendency to prioritize the need for efficiency or safety in emergency dispatch further validates the applicability of drones in high perceived usefulness scenarios.

From the regression results in Table 6, the independent variables with Sig. values less than 0.05 had a significant effect on S_4._ These independent variables include the following: V_4_ (does your hospital have/experiment with drone delivery), V_5_ (how often does your hospital urgently dispatch medical supplies from other places), V8 (the efficiency of your current hospital logistics department), V_10_ (What do you think about the cost of drone delivery), V_11_ (whether you think it is safe to deliver medicines by drone), and V_14_ (whether there is more policy support for drone delivery in your region). In particular, the efficiency of the hospital logistics sector, as an emerging influence, reflects the magnitude of the relative advantage of drones. If existing logistics are highly efficient, the relative improvement in efficiency provided by drones is small, with a corresponding decrease in perceived usefulness. On the other hand, if traditional methods are less efficient, drones provide more significant efficiency improvements and perceived usefulness rises. The other factors are consistent with the previous lines of analysis.

From the final analysis results of the four independent variable models, the factors that had a significant impact on the dependent variable were different, and this result was due to the different characteristics of the distribution materials themselves. In general, there was a trend that the lower the value and scarcity of materials, the fewer independent variables could cause a significant impact. Based on the properties of the binary logistic model (40), if a method could be found in the future to improve medical personnel’s evaluations of the above independent variables (which could cause significant impacts), it would theoretically lead to an increase in their willingness to adopt drone delivery. Considering that it was impractical to differentiate vehicle models, management methods, or delivery routes in real-world drone medical supply delivery scenarios, future studies would integrate all significant independent variables across the four dependent variable models to derive comprehensive conclusions.

### 5.2 Fuzzy-ISM analysis

#### 5.2.1 Identifying deep influencing factors

**Figure 2:**
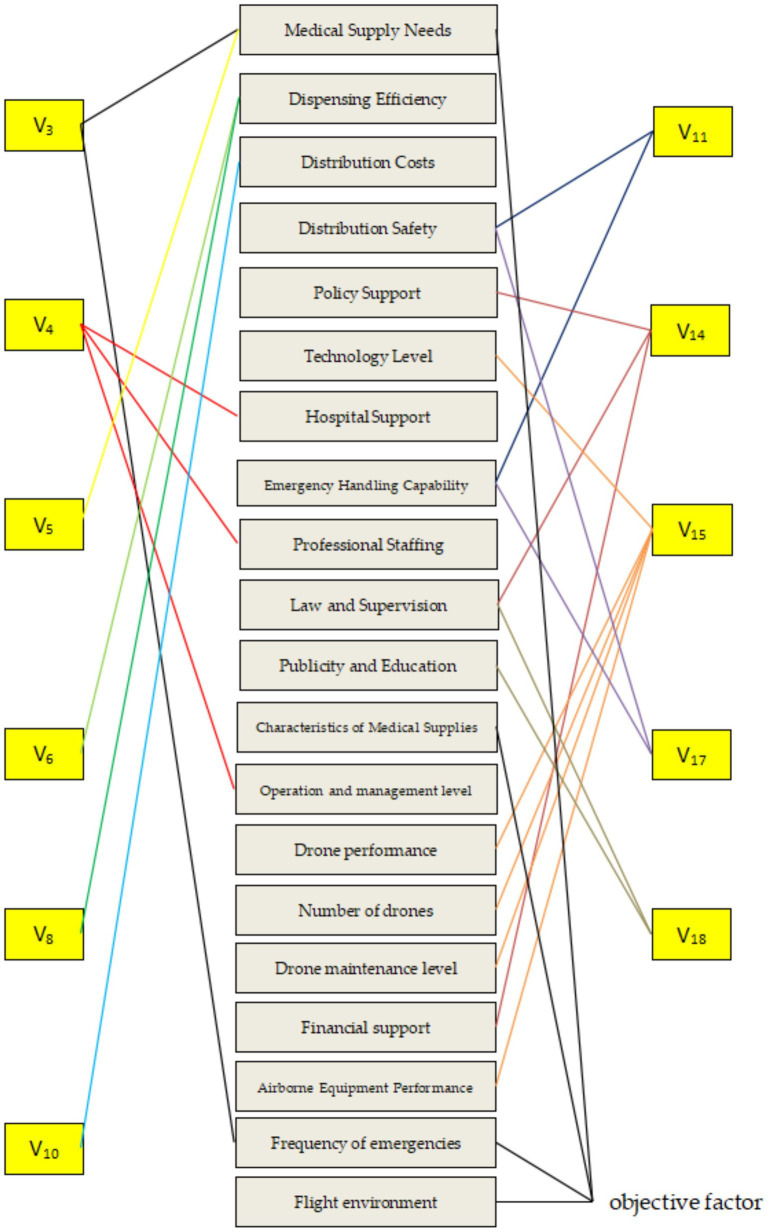
Correspondence map between causes and deep-rooted factors.

Using the binary logistic model analysis, we identified the reasons that can directly affect the willingness of medical workers to use drones, and theoretically only need to make the medical workers’ evaluation of these reasons to be changed, can directly improve the medical workers’ existing concerns. This is to deeply analyze whether there are deeper logical connections between these reasons and to use scientific means to determine the priorities and focuses of future drone delivery of medical supplies activities. Based on the previous descriptive analysis, the team’s previous research experience and literature references (27–37), the representative deeper factors inherent in these causes were identified, and the results are shown in Figure 2, and the deeper factors are numbered and briefly described for the convenience of subsequent research (Table 7).

**Table 7:** Deep factor numbering and short description table.

No.	Name of factor	Brief description of content
F_1_	Medical supply needs	Medical personnel need to use off-site drones to marshal medical supply needs
F_2_	Dispensing efficiency	Efficiency of Drone Distribution of Medical Supplies
F_3_	Distribution costs	Overall cost of drone medical supply distribution activities
F_4_	Distribution safety	Safety of Drone Delivery of Medical Supplies
F_5_	Policy support	Local Policies for Drone Delivery of Medical Supplies
F_6_	Technology level	Current level of drone delivery technology
F_7_	Hospital support	Hospital support for drone delivery
F_8_	Emergency handling capability	Emergency response capabilities during the campaign
F_9_	Professional staffing	Quality and quantity of specialized personnel in all aspects of the activity
F_10_	Law and supervision	Legal supervision and management of drone activities
F_11_	Publicity and education	Efforts to publicize and educate the public about drone delivery
F_12_	Characteristics of medical supplies	Physical and chemical properties of the medical materials to be delivered, etc.
F_13_	Operation and management level	Overall operational quality and management capability
F_14_	Drone performance	Comprehensive performance of the drones used for delivery
F_15_	Number of drones	The overall number of drones for the campaign
F_16_	Drone maintenance level	The level of maintenance and investment in the drones on a daily basis
F_17_	Financial support	Overall financial support for the drone delivery program.
F_18_	Airborne Equipment Performance	Efficacy of special equipment and loads on board, etc.
F_19_	Frequency of emergencies	Frequency of emergencies that require the drone to make emergency deliveries.
F_20_	Flight environment	The flying environment of the area where the drone is located

#### 5.2.2 Data processing

As there may be a relationship of some direct or indirect influence between the above deep factors, the interrelationship between these factors can be effectively determined by utilizing the Fuzzy-ISM described in Part III. The Fuzzy-ISM established through multi-expert scoring can largely avoid the problems of overly subjective analysis that exist in personal evaluation. The use of certain mathematical methods in the synthesis of multi-expert scoring makes the constructed model highly accurate and logical and does not require repeated adjustments to the final model, as in the case of the traditional ISM method (46).

Five options made up the rating scale given to the experts, each of which represented the level of evaluation of a factor on the comparison factor. Following the collection of the rating scale, the evaluation text was transformed into numerical scoring values. Table 8 displays the criteria. Table 9 displays the primary matrix table created with MATLAB based on the data characteristics for the weighted average of the collected data. The values show the weighted average of the degree to which the corresponding vertical factors have an impact on the horizontal factors. After obtaining the primary matrix, the ISM model characteristics and the principles of fuzzy mathematics are combined to find the strength of association matrix, and the transformation formula Equation (2) is as follows:


(2) $$G_{ij}=\frac{C_{ij}}{C_{i•}+C_{•j}-C_{ij}}$$

**Table 8:** Criteria for evaluating the degree of influence of deep-seated factors.

Level options	No or minimal impact	Low impact	Medium impact	High impact	Deep Impact
Score	0	0.25	0.5	0.75	1

**Table 9:** Primary matrix data table for deep factors ISM analysis (C).

Factor number	F_1_	F_2_	F_3_	F_4_	F_5_	F_6_	F_7_	F_8_	F_9_	F_10_	F_11_	F_12_	F_13_	F_14_	F_15_	F_16_	F_17_	F_18_	F_19_	F_20_
F_1_	0	0.171428571	0.692857143	0.157142857	0.121428571	0.2	0.171428571	0.171428571	0.685714286	0.178571429	0.292857143	0.207142857	0.121428571	0.157142857	0.7	0.214285714	0.185714286	0.114285714	0.221428571	0.221428571
F_2_	0.164285714	0	0.228571429	0.142857143	0.178571429	0.107142857	0.257142857	0.15	0.114285714	0.214285714	0.192857143	0.171428571	0.25	0.2	0.192857143	0.242857143	0.121428571	0.278571429	0.142857143	0.135714286
F_3_	0.192857143	0.278571429	0	0.135714286	0.157142857	0.242857143	0.185714286	0.135714286	0.157142857	0.157142857	0.157142857	0.171428571	0.764285714	0.271428571	0.164285714	0.25	0.178571429	0.135714286	0.228571429	0.121428571
F_4_	0.192857143	0.828571429	0.7	0	0.228571429	0.171428571	0.228571429	0.15	0.207142857	0.207142857	0.157142857	0.178571429	0.257142857	0.185714286	0.221428571	0.171428571	0.207142857	0.257142857	0.142857143	0.135714286
F_5_	0.107142857	0.142857143	0.1	0.185714286	0	0.157142857	0.8	0.185714286	0.2	0.721428571	0.685714286	0.235714286	0.192857143	0.135714286	0.221428571	0.15	0.671428571	0.214285714	0.185714286	0.292857143
F_6_	0.221428571	0.785714286	0.85	0.892857143	0.142857143	0	0.221428571	0.171428571	0.771428571	0.292857143	0.192857143	0.171428571	0.192857143	0.742857143	0.792857143	0.742857143	0.264285714	0.792857143	0.235714286	0.107142857
F_7_	0.2	0.792857143	0.128571429	0.25	0.164285714	0.142857143	0	0.121428571	0.792857143	0.221428571	0.164285714	0.185714286	0.8	0.235714286	0.778571429	0.807142857	0.678571429	0.2	0.164285714	0.207142857
F_8_	0.214285714	0.828571429	0.857142857	0.835714286	0.192857143	0.214285714	0.192857143	0	0.164285714	0.178571429	0.135714286	0.242857143	0.171428571	0.128571429	0.207142857	0.15	0.121428571	0.207142857	0.128571429	0.171428571
F_9_	0.178571429	0.807142857	0.892857143	0.192857143	0.171428571	0.128571429	0.078571429	0.792857143	0	0.164285714	0.171428571	0.157142857	0.821428571	0.2	0.278571429	0.8	0.185714286	0.121428571	0.235714286	0.1
F_10_	0.05	0.1	0.121428571	0.785714286	0.178571429	0.142857143	0.121428571	0.142857143	0.121428571	0	0.8	0.2	0.792857143	0.142857143	0.257142857	0.207142857	0.207142857	0.178571429	0.135714286	0.214285714
F_11_	0.078571429	0.071428571	0.157142857	0.207142857	0.821428571	0.121428571	0.85	0.114285714	0.164285714	0.171428571	0	0.128571429	0.121428571	0.207142857	0.171428571	0.164285714	0.135714286	0.107142857	0.157142857	0.142857143
F_12_	0.207142857	0.792857143	0.85	0.2	0.171428571	0.142857143	0.142857143	0.142857143	0.092857143	0.114285714	0.142857143	0	0.142857143	0.835714286	0.192857143	0.142857143	0.042857143	0.978571429	0.178571429	0.242857143
F_13_	0.092857143	0.792857143	0.857142857	0.814285714	0.2	0.157142857	0.171428571	0.792857143	0.164285714	0.192857143	0.142857143	0.171428571	0	0.128571429	0.278571429	0.235714286	0.171428571	0.171428571	0.114285714	0.1
F_14_	0.142857143	0.814285714	0.75	0.778571429	0.221428571	0.171428571	0.185714286	0.121428571	0.192857143	0.157142857	0.157142857	0.128571429	0.142857143	0	0.8	0.792857143	0.157142857	0.2	0.2	0.178571429
F_15_	0.192857143	0.792857143	0.842857143	0.192857143	0.171428571	0.2	0.107142857	0.257142857	0.764285714	0.221428571	0.221428571	0.185714286	0.157142857	0.185714286	0	0.864285714	0.185714286	0.171428571	0.157142857	0.135714286
F_16_	0.185714286	0.814285714	0.807142857	0.85	0.1	0.192857143	0.192857143	0.157142857	0.121428571	0.2	0.171428571	0.121428571	0.207142857	0.15	0.207142857	0	0.185714286	0.142857143	0.121428571	0.185714286
F_17_	0.192857143	0.157142857	0.25	0.157142857	0.235714286	0.757142857	0.25	0.2	0.8	0.2	0.2	0.207142857	0.857142857	0.814285714	0.785714286	0.842857143	0	0.792857143	0.185714286	0.157142857
F_18_	0.157142857	0.842857143	0.857142857	0.828571429	0.121428571	0.085714286	0.121428571	0.178571429	0.828571429	0.192857143	0.207142857	0.135714286	0.221428571	0.135714286	0.157142857	0.8	0.121428571	0	0.164285714	0.142857143
F_19_	0.892857143	0.135714286	0.178571429	0.135714286	0.842857143	0.221428571	0.807142857	0.15	0.121428571	0.164285714	0.171428571	0.828571429	0.178571429	0.192857143	0.185714286	0.164285714	0.842857143	0.221428571	0	0.192857143
F_20_	0.185714286	0.85	0.907142857	0.835714286	0.728571429	0.828571429	0.142857143	0.171428571	0.157142857	0.185714286	0.157142857	0.15	0.192857143	0.192857143	0.814285714	0.807142857	0.192857143	0.807142857	0.185714286	0

Where G_ij_ is the factor in the j-th column of the i-th row in the association strength matrix, C_-ij_ is the factor in the j-th column of the i-th row in the initial matrix, C_i._ is the sum of the values of the factors in the ith row in the initial matrix, and C_.j_ is the sum of the values of the factors in the j-th column in the initial matrix. The data for generating the strength of association matrix using MATLAB is shown in Table 10.

**Table 10:** Data table for matrix of strength of association of deep factors (G).

Factor number	F_1_	F_2_	F_3_	F_4_	F_5_	F_6_	F_7_	F_8_	F_9_	F_10_	F_11_	F_12_	F_13_	F_14_	F_15_	F_16_	F_17_	F_18_	F_19_	F_20_
F_1_	0	0.010978957	0.045221445	0.011720831	0.012125535	0.021806854	0.017069701	0.018794049	0.062786135	0.019968051	0.031782946	0.02365416	0.010605115	0.015602837	0.059865608	0.016085791	0.019230769	0.010423453	0.027506655	0.02785265
F_2_	0.022908367	0	0.016	0.011983223	0.021114865	0.013799448	0.030405405	0.019626168	0.011436741	0.028929605	0.024680073	0.023506366	0.025454545	0.023450586	0.018024032	0.02059358	0.014769765	0.029953917	0.021551724	0.020765027
F_3_	0.024907749	0.019070905	0	0.010832383	0.017309205	0.029513889	0.020344288	0.016435986	0.014895058	0.019486271	0.018596788	0.021719457	0.077144917	0.029968454	0.014501892	0.020184544	0.020374898	0.013513514	0.032	0.016983017
F_4_	0.022727273	0.055984556	0.046182846	0	0.023443223	0.018957346	0.023255814	0.016693164	0.018424396	0.02365416	0.017094017	0.020695364	0.023047375	0.018786127	0.01843044	0.012979989	0.021853806	0.024112525	0.017921147	0.017225748
F_5_	0.011485452	0.008795075	0.006055363	0.013285641	0	0.016011645	0.079885877	0.019131714	0.016656752	0.08015873	0.072782411	0.025267994	0.016100179	0.012692051	0.017337808	0.01072523	0.06871345	0.018691589	0.021381579	0.034540859
F_6_	0.018128655	0.042242704	0.04529882	0.054872695	0.010509721	0	0.016290068	0.013475576	0.053438892	0.023563218	0.014933628	0.013832853	0.012875536	0.056768559	0.052161654	0.045315904	0.020054201	0.057098765	0.020257827	0.009185548
F_7_	0.018716578	0.046521375	0.007168459	0.016271502	0.013666072	0.012666244	0	0.010821133	0.061632426	0.020221787	0.014420063	0.017150396	0.062395543	0.019572954	0.056978568	0.054615756	0.060509554	0.015469613	0.016174402	0.020684736
F_8_	0.023866348	0.054104478	0.055248619	0.063864629	0.018723994	0.022522523	0.018582244	0	0.013922518	0.019201229	0.013950073	0.02675059	0.014580802	0.012295082	0.016514806	0.010914761	0.012048193	0.018447837	0.01512605	0.020512821
F_9_	0.017593244	0.049002602	0.053740327	0.012974531	0.014962594	0.011976048	0.006756757	0.079342387	0	0.015721121	0.015831135	0.015256588	0.067094516	0.017358958	0.020472441	0.0562249	0.01665599	0.009753299	0.024737631	0.010455564
F_10_	0.005747126	0.006410256	0.00768188	0.061902082	0.018089725	0.015625	0.01213419	0.015760441	0.010651629	0	0.092792046	0.023045267	0.074148297	0.014285714	0.021339656	0.015641855	0.021690352	0.01651255	0.016858917	0.02722323
F_11_	0.009990917	0.004819277	0.010501193	0.016618911	0.097540288	0.014529915	0.100337268	0.013793103	0.015572106	0.021276596	0	0.01618705	0.01150203	0.022691706	0.015132409	0.013165426	0.015397083	0.010630758	0.021760633	0.02002002
F_12_	0.022036474	0.050294517	0.05333931	0.01414856	0.015968064	0.014285714	0.013175231	0.014398848	0.00755814	0.011687363	0.014094433	0	0.011709602	0.082220661	0.014867841	0.010085729	0.004054054	0.090013141	0.020145044	0.027914614
F_13_	0.009767092	0.050317316	0.053835801	0.0602537	0.018691589	0.015748031	0.015862525	0.085582113	0.013458163	0.019896831	0.014104372	0.01793722	0	0.01183432	0.021630616	0.016759777	0.016427105	0.014687882	0.012810248	0.011317704
F_14_	0.014285714	0.050021939	0.045258621	0.055245819	0.019732654	0.016315432	0.016383113	0.011588275	0.015160022	0.015299026	0.014745308	0.012676056	0.01121705	0	0.062015504	0.056431113	0.014294997	0.016412661	0.021325209	0.019201229
F_15_	0.019955654	0.049509367	0.052051169	0.013399504	0.015604681	0.019621584	0.009627728	0.025568182	0.064419025	0.022318215	0.021483021	0.018950437	0.012636416	0.016785023	0	0.063119457	0.017391304	0.014371257	0.017200938	0.014984227
F_16_	0.021155411	0.053926206	0.052631579	0.06618465	0.00983837	0.020721412	0.019000704	0.016962221	0.010455105	0.022099448	0.018113208	0.013535032	0.018023617	0.014695591	0.016821346	0	0.018978102	0.012911556	0.014667817	0.022887324
F_17_	0.016483516	0.008409786	0.013282732	0.009544469	0.018191841	0.064871481	0.019199122	0.016460905	0.057702215	0.016696482	0.016175621	0.017533253	0.062240664	0.065292096	0.053580127	0.053514739	0	0.059421842	0.016666667	0.014193548
F_18_	0.015725518	0.051845343	0.052038161	0.058973055	0.010718789	0.008086253	0.010644959	0.017123288	0.068517425	0.018828452	0.019515478	0.013380282	0.01748449	0.011897307	0.011597259	0.056939502	0.011003236	0	0.017437453	0.01529052
F_19_	0.09314456	0.007847997	0.010216592	0.009004739	0.077073808	0.020516214	0.073044602	0.013907285	0.009249184	0.015498652	0.015614834	0.084733382	0.01369863	0.016513761	0.013408974	0.01094196	0.079194631	0.017714286	0	0.020044543
F_20_	0.015276146	0.046088304	0.048733691	0.051473823	0.056415929	0.068761114	0.010520779	0.013574661	0.010506208	0.014925373	0.012222222	0.012173913	0.012955854	0.014240506	0.053977273	0.049714034	0.01465798	0.058579575	0.016019717	0

#### 5.2.3 Determination of model adjacency matrix

To facilitate the subsequent data analysis and ISM modeling work, after obtaining the correlation strength matrix by judging the correlation strength matrix value, the set threshold relationship can be converted to the traditional ISM, as shown in the basic multi-order matrix model. Through exchanges with experts and experience judgment, that the threshold value of 0.045 (considering the analysis process there is a small error or cause the existence of some of the factors have a certain impact on the misjudgment of the degree of existence of the factors, it is chosen to be slightly lower than the theoretical median value of the value), and is converted into an adjacency matrix using Equation (3):


(3) $$A_{ij}={\{}_{0,Gij<0.045}^{1,Gij\geq 0.045}$$

Where A_ij_ is the value of the factors in row i and column j of the adjacency matrix. At this point, MATLAB was used to calculate the adjacency matrix for this study, which can be directly used for subsequent ISM modeling:

#### 5.2.4 Establish the model reachable matrix

This is based on the nature of Boolean matrix operations on the adjacency matrix A and the unit matrix I (this study for the 20th order) for a number of power operations, for example, when to meet the B = (A + I)^n^ = (A + I)^n + 1^ ≠ (A + I)^n-1^ to stop calculating, this time to find the reachable matrix B. In the numerical significance of the reachable matrix, the element 1 indicated that there is a stronger logical relationship between the factors to own the reachable path; the element 0 indicated that between the two factors element 0 indicated that there is no strong logical connection between the two factors. It is necessary to use MATLAB programming to help with the computation of the power of four operations in order to study the reachable matrix because of the intricacy of the arithmetic process and the volume of data.

#### 5.2.5 Hierarchical decomposition and determination of multilevel structural diagrams

By hierarchical processing of the reachability matrix, we can achieve a more systematic and intuitive understanding of the existence of the logical relationship between the factors. In the reachable matrix, the set of factors influenced by F_i_ forms the reachable set P = (F_i_), and the set of factors influencing I_i_ forms the prior set Q = (F_i_); the intersection operation between the reachable set and the prior set is carried out, and in the case of P = (F_i_) = P(F_i_)∩Q(F_i_), the uppermost factor is F_i_; and after that, a new reachable matrix can be formed by crossing out the rows and columns in which it is located; Repeat the above hierarchical decomposition steps several times to divide the layers of the final model and their corresponding factors. The results of the hierarchical decomposition (from top to bottom) are shown in Table 11.

**Table 11:** Hierarchical decomposition results.

Number of layers	Factors
First layer	F_2_
Second layer	F_3_, F_4_, F_8_, F_13_
Third Layer	F_16_
Fourth layer	F_9_
Fifth layer	F_15_, F_18_
Sixth layer	F_1_, F_14_
Seventh layer	F_6_, F_12_
Eighth layer	F_17_
Ninth layer	F_7_
Tenth layer	F_5_, F_10_, F_11_
Eleventh layer	F_19_, F_20_

**Figure 3:**
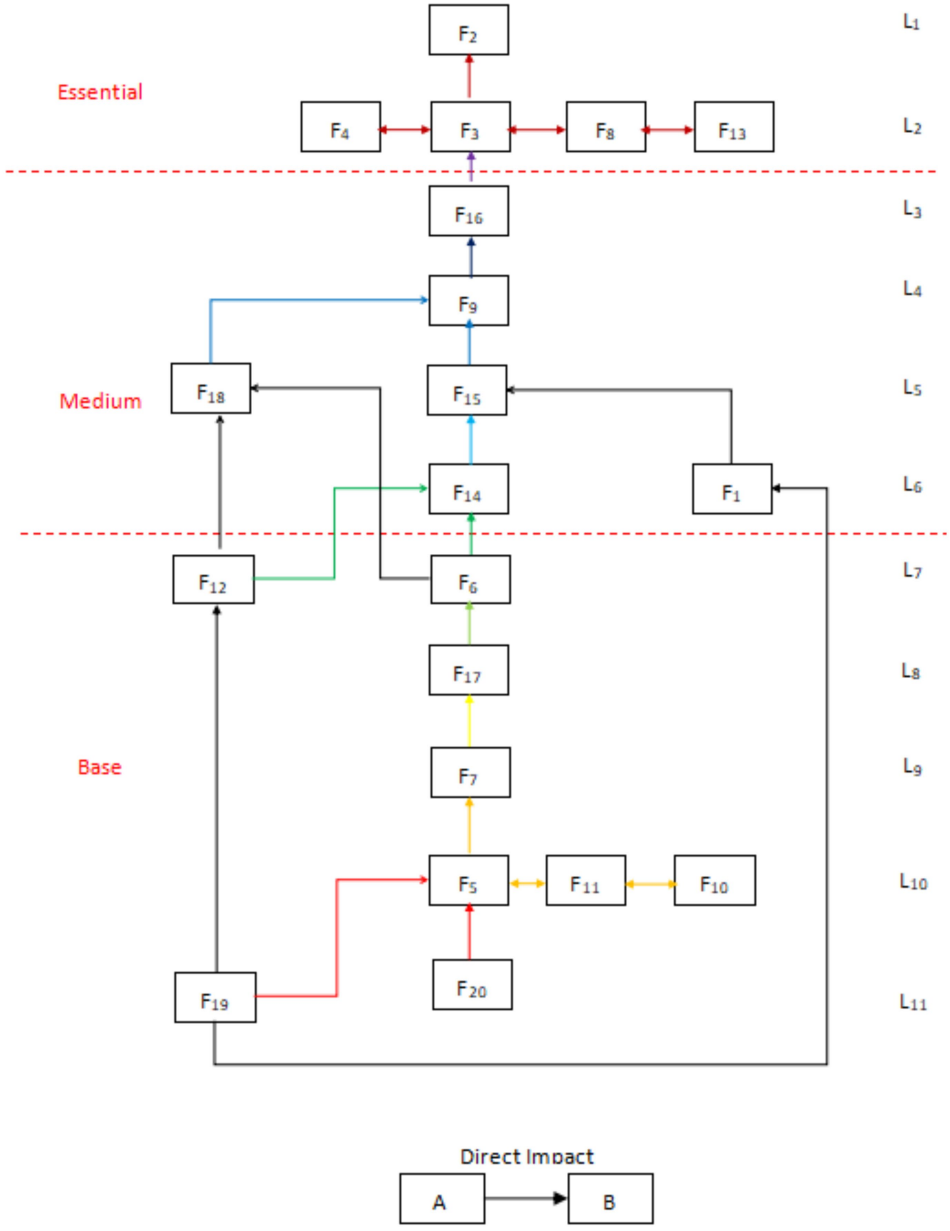
ISM multi-level conclusion structure diagram.

**Figure 4:**
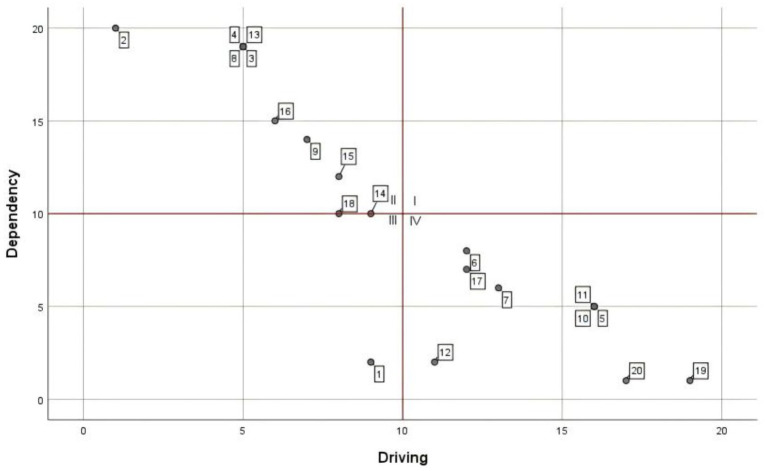
Distribution of MICMAC coordinates.

According to the mutual influence relationship between the factors at each level and other factors, the ISM multilevel structure diagram is made, see Figure 3. In addition, in order to deepen the systematic research, the number of factors influencing and being influenced in the reachability matrix is used to perform the driving dependence analysis, and the coordinate distribution diagram of the factor cross-influence matrix multiplication method (MICMAC) is made, as shown in Figure 4.

#### 5.2.6 Model interpretation

According to the principle of the ISM method (46), combining the factors’ characteristics with the ISM multilevel conclusion structure diagram and MICMAC coordinate diagram, the factors located in the 7th to 11th layers had high drive and low dependence. They were located in the bottom layer of ISM, which indicated that these factors are the basis for constructing the evaluation of the use of drones by medical workers in the delivery of medical supplies. From the Technology Acceptance Model (TAM) perspective, these factors critically support the perceived ease of use (PEOU) by addressing systemic usability barriers. Simultaneously, the (DOI) framework highlights their dual function in ensuring compatibility with existing institutional protocols and enabling trialability through pilot validations—key prerequisites for scalable technology adoption. The construction of these factors was indispensable, but a single increase in the overall activity of this part will not effectively alleviate medical workers’ concerns. In terms of the nature of these factors, apart from the frequency of emergencies, flight environment, and the characteristics of medical supplies, which were objective factors, the remaining elements including technical level, financial support, hospital support, policy support, law and regulation, and publicity and education all belonged to the developmental environment in the construction of drone delivery of medical supplies. The construction of these factors must be carried out throughout all the periods of the activity to create a good developmental environment for the subsequent and better evaluation of medical supplies. A good development environment was conducive to the subsequent promotion of the drone delivery of medical supplies program.

The factors located in layers 3 to 6—with the exception of the demand for medical supplies, which was an objective factor—had low driving force and high dependence. These remaining elements, encompassing drone maintenance level, professional staffing, drone quantity, airborne equipment performance, and drone performance characteristics, were all categorized as infrastructure components in the operation of drone medical delivery systems based on their functional attributes. These factors determine the feasibility and difficulty of running the system and have a direct impact on perceived ease of use (PEOU). The better the infrastructure, the easier the system is to use and the higher the PEOU; when the infrastructure is inadequate, the complexity of the project increases and so does the resistance to use. At the same time, the optimization of these mid-level factors enhances the testability and reduces the complexity of the technology, creating conditions for subsequent pilot roll-outs. From the MICMAC chart, these factors had a high dependence, which indicated that strengthening the construction of the relevant content of these factors could effectively improve the willingness of medical personnel to use drones to distribute medical materials. According to the questionnaire data, medical personnel in hospitals with prior experience in drone delivery—including those familiar with drone operation principles, having conducted trial programs, or maintaining active drone services—showed a higher propensity to utilize drones for various medical material deliveries compared to their counterparts in non-practicing institutions.

This discrepancy indicated that improving the construction of this part of the project can effectively change the concepts of medical personnel.

Factors in layers 1 and 2 are as follows: dispensing efficiency, distribution cost, distribution safety, emergency handling capability, and operation and management level. These all had extremely high dependency and low driving force, indicating that the willingness of medical workers to use drones could be greatly improved by handling these factors well. According to TAM, these factors significantly influence the perceived usefulness (PU) of a system. For example, efficiency or security gains enhance the user’s perception of usefulness. From a DOI perspective, they reflect the relative advantage of the innovation and are key indicators that determine the eventual adoption of the technology. Based on the development status of drone delivery for medical supplies at the time of the study, no region or organization had achieved regular operation, and most initiatives were in the experimental stage. It was recommended that the optimization of this part of the factors be actively considered in the experimental stage so as to ensure that a set of more mature operational solutions could be used in future large-scale applications. Regarding current progress in drone delivery development, although most countries or regions maintain an encouraging attitude, from the overall construction progress, the realization of normalized operation might still need to wait a very long time. Through the improvement of these factors related to the realization of the overall level of willingness of medical workers to use, this enhancement might also need to be validated only after many years. From existing research (23), the use of drones to distribute materials on the technical level was no longer prominent. How to effectively solve the related management problems in the future might become the focus of the next stage of research.


$$A=\left[\begin{array}{ccccccccccccccccccccc} & F_{1} & F_{2} & F_{3} & F_{4} & F_{5} & F_{6} & F_{7} & F_{8} & F_{9} & F_{10} & F_{11} & F_{12} & F_{13} & F_{14} & F_{15} & F_{16} & F_{17} & F_{18} & F_{19} & F_{20}\\ F_{1} & 0 & 0 & 1 & 0 & 0 & 0 & 0 & 0 & 1 & 0 & 0 & 0 & 0 & 0 & 1 & 0 & 0 & 0 & 0 & 0\\ F_{2} & 0 & 0 & 0 & 0 & 0 & 0 & 0 & 0 & 0 & 0 & 0 & 0 & 0 & 0 & 0 & 0 & 0 & 0 & 0 & 0\\ F_{3} & 0 & 0 & 0 & 0 & 0 & 0 & 0 & 0 & 0 & 0 & 0 & 0 & 1 & 0 & 0 & 0 & 0 & 0 & 0 & 0\\ F_{4} & 0 & 1 & 1 & 0 & 0 & 0 & 0 & 0 & 0 & 0 & 0 & 0 & 0 & 0 & 0 & 0 & 0 & 0 & 0 & 0\\ F_{5} & 0 & 0 & 0 & 0 & 0 & 0 & 1 & 0 & 0 & 1 & 1 & 0 & 0 & 0 & 0 & 0 & 1 & 0 & 0 & 0\\ F_{6} & 0 & 1 & 1 & 1 & 0 & 0 & 0 & 0 & 1 & 0 & 0 & 0 & 0 & 1 & 1 & 1 & 0 & 1 & 0 & 0\\ F_{7} & 0 & 1 & 0 & 0 & 0 & 0 & 0 & 0 & 1 & 0 & 0 & 0 & 1 & 0 & 1 & 1 & 1 & 0 & 0 & 0\\ F_{8} & 0 & 1 & 1 & 1 & 0 & 0 & 0 & 0 & 0 & 0 & 0 & 0 & 0 & 0 & 0 & 0 & 0 & 0 & 0 & 0\\ F_{9} & 0 & 1 & 1 & 0 & 0 & 0 & 0 & 1 & 0 & 0 & 0 & 0 & 1 & 0 & 0 & 1 & 0 & 0 & 0 & 0\\ F_{10} & 0 & 0 & 0 & 1 & 0 & 0 & 0 & 0 & 0 & 0 & 1 & 0 & 1 & 0 & 0 & 0 & 0 & 0 & 0 & 0\\ F_{11} & 0 & 0 & 0 & 0 & 1 & 0 & 1 & 0 & 0 & 0 & 0 & 0 & 0 & 0 & 0 & 0 & 0 & 0 & 0 & 0\\ F_{12} & 0 & 1 & 1 & 0 & 0 & 0 & 0 & 0 & 0 & 0 & 0 & 0 & 0 & 1 & 0 & 0 & 0 & 1 & 0 & 0\\ F_{13} & 0 & 1 & 1 & 1 & 0 & 0 & 0 & 1 & 0 & 0 & 0 & 0 & 0 & 0 & 0 & 0 & 0 & 0 & 0 & 0\\ F_{14} & 0 & 1 & 1 & 1 & 0 & 0 & 0 & 0 & 0 & 0 & 0 & 0 & 0 & 0 & 1 & 1 & 0 & 0 & 0 & 0\\ F_{15} & 0 & 1 & 1 & 0 & 0 & 0 & 0 & 0 & 1 & 0 & 0 & 0 & 0 & 0 & 0 & 1 & 0 & 0 & 0 & 0\\ F_{16} & 0 & 1 & 1 & 1 & 0 & 0 & 0 & 0 & 0 & 0 & 0 & 0 & 0 & 0 & 0 & 0 & 0 & 0 & 0 & 0\\ F_{17} & 0 & 0 & 0 & 0 & 0 & 1 & 0 & 1 & 0 & 0 & 0 & 0 & 1 & 1 & 1 & 1 & 0 & 1 & 0 & 0\\ F_{18} & 0 & 1 & 1 & 1 & 0 & 0 & 0 & 0 & 1 & 0 & 0 & 0 & 0 & 0 & 0 & 1 & 0 & 0 & 0 & 0\\ F_{19} & 1 & 0 & 0 & 0 & 1 & 0 & 1 & 0 & 0 & 0 & 0 & 1 & 0 & 0 & 0 & 0 & 1 & 0 & 0 & 0\\ F_{20} & 0 & 1 & 1 & 1 & 1 & 1 & 0 & 0 & 0 & 0 & 0 & 0 & 0 & 0 & 1 & 1 & 0 & 1 & 0 & 0 \end{array}\right]$$

$$B=\left[\begin{array}{ccccccccccccccccccccc} & F_{1} & F_{2} & F_{3} & F_{4} & F_{5} & F_{6} & F_{7} & F_{8} & F_{9} & F_{10} & F_{11} & F_{12} & F_{13} & F_{14} & F_{15} & F_{16} & F_{17} & F_{18} & F_{19} & F_{20}\\ F_{1} & 1 & 1 & 1 & 1 & 0 & 0 & 0 & 1 & 1 & 0 & 0 & 0 & 1 & 0 & 1 & 1 & 0 & 0 & 0 & 0\\ F_{2} & 0 & 1 & 0 & 0 & 0 & 0 & 0 & 0 & 0 & 0 & 0 & 0 & 0 & 0 & 0 & 0 & 0 & 0 & 0 & 0\\ F_{3} & 0 & 1 & 1 & 1 & 0 & 0 & 0 & 1 & 0 & 0 & 0 & 0 & 1 & 0 & 0 & 0 & 0 & 0 & 0 & 0\\ F_{4} & 0 & 1 & 1 & 1 & 0 & 0 & 0 & 1 & 0 & 0 & 0 & 0 & 1 & 0 & 0 & 0 & 0 & 0 & 0 & 0\\ F_{5} & 0 & 1 & 1 & 1 & 1 & 1 & 1 & 1 & 1 & 1 & 1 & 0 & 1 & 1 & 1 & 1 & 1 & 1 & 0 & 0\\ F_{6} & 0 & 1 & 1 & 1 & 0 & 1 & 0 & 1 & 1 & 0 & 0 & 0 & 1 & 1 & 1 & 1 & 0 & 1 & 0 & 0\\ F_{7} & 0 & 1 & 1 & 1 & 0 & 1 & 1 & 1 & 1 & 0 & 0 & 0 & 1 & 1 & 1 & 1 & 1 & 1 & 0 & 0\\ F_{8} & 0 & 1 & 1 & 1 & 0 & 0 & 0 & 1 & 0 & 0 & 0 & 0 & 1 & 0 & 0 & 0 & 0 & 0 & 0 & 0\\ F_{9} & 0 & 1 & 1 & 1 & 0 & 0 & 0 & 1 & 1 & 0 & 0 & 0 & 1 & 0 & 0 & 1 & 0 & 0 & 0 & 0\\ F_{10} & 0 & 1 & 1 & 1 & 1 & 1 & 1 & 1 & 1 & 1 & 1 & 0 & 1 & 1 & 1 & 1 & 1 & 1 & 0 & 0\\ F_{11} & 0 & 1 & 1 & 1 & 1 & 1 & 1 & 1 & 1 & 1 & 1 & 0 & 1 & 1 & 1 & 1 & 1 & 1 & 0 & 0\\ F_{12} & 0 & 1 & 1 & 1 & 0 & 0 & 0 & 1 & 1 & 0 & 0 & 1 & 1 & 1 & 1 & 1 & 0 & 1 & 0 & 0\\ F_{13} & 0 & 1 & 1 & 1 & 0 & 0 & 0 & 1 & 0 & 0 & 0 & 0 & 1 & 0 & 0 & 0 & 0 & 0 & 0 & 0\\ F_{14} & 0 & 1 & 1 & 1 & 0 & 0 & 0 & 1 & 1 & 0 & 0 & 0 & 1 & 1 & 1 & 1 & 0 & 0 & 0 & 0\\ F_{15} & 0 & 1 & 1 & 1 & 0 & 0 & 0 & 1 & 1 & 0 & 0 & 0 & 1 & 0 & 1 & 1 & 0 & 0 & 0 & 0\\ F_{16} & 0 & 1 & 1 & 1 & 0 & 0 & 0 & 1 & 0 & 0 & 0 & 0 & 1 & 0 & 0 & 1 & 0 & 0 & 0 & 0\\ F_{17} & 0 & 1 & 1 & 1 & 0 & 1 & 0 & 1 & 1 & 0 & 0 & 0 & 1 & 1 & 1 & 1 & 1 & 1 & 0 & 0\\ F_{18} & 0 & 1 & 1 & 1 & 0 & 0 & 0 & 1 & 1 & 0 & 0 & 0 & 1 & 0 & 0 & 1 & 0 & 1 & 0 & 0\\ F_{19} & 1 & 1 & 1 & 1 & 1 & 1 & 1 & 1 & 1 & 1 & 1 & 1 & 1 & 1 & 1 & 1 & 1 & 1 & 1 & 0\\ F_{20} & 0 & 1 & 1 & 1 & 1 & 1 & 1 & 1 & 1 & 1 & 1 & 0 & 1 & 1 & 1 & 1 & 1 & 1 & 0 & 1 \end{array}\right]$$

## 6 Discussion

The three-stage model of “infrastructure construction-technology performance optimization-operational efficiency enhancement” constructed in this study echoes the theory of increasing marginal benefits proposed by Johannessen (38) in terms of methodology but breaks away from its analytical framework, focusing on a single economic dimension. Compared to the research paths of Bhatt et al. (34) and Nisingizwe et al. (35), which focus on the validation of efficiency in specific scenarios, the present model integrates the human element constraints on technology adoption, which is theoretically complementary to the cognitive characteristics of medical personnel found by Sham (46). This study also explains the current dilemma of medical drone diffusion: most of the projects are stagnant in the transitional stage of institutional safeguard and technology validation (corresponding to the “knowledge-persuasion” period of innovation diffusion) and have not yet formed a complete value creation chain.

In this study, the key factors affecting medical personnel’s willingness to use drones to deliver medical supplies and their inherent logical relationships are clearly analyzed. Referring to the results of the previous analyses, from a systemic point of view, it is necessary to prioritize the construction of high-driven factors to ensure that the normal operation of the project can be met, and then strengthen the construction of high-dependency factors as much as possible, in order to increase the willingness of medical personnel to use drones to deliver medical supplies more effectively. Based on this, this study concludes that the following.

For projects that are still in the early stages of construction, it is inappropriate to rush to promote the rapid acceptance of the emerging model of drone delivery by medical personnel. Instead, the focus at this stage should be on preliminary preparatory work to create a favorable development environment for the project. Specific recommendations include optimizing the existing flight environment, strengthening policy support, improving the legal monitoring mechanism, increasing publicity and financial investment, introducing advanced technologies, and raising the importance of hospitals to relevant technologies. The construction focus of this stage covers factors F_5_, F_6_, F_7_, F_10_, F_11_, and F_17_ (due to the special nature of medical materials and the frequency of emergencies, objective factors such as F_12_ and F_19_ are not included in the optimization scope of this study). Ensuring the long-term stability of the relevant work will lay a solid foundation for the sustainable development of the UAV medical delivery program. In contrast to existing literature that analyses cost-effectiveness (38, 39), this study deepens the cost–time substitution theory proposed by Zailani et al. (40) through the cost factor (F10) transmission mechanism revealed by the multifactor interaction model. Especially in the emergency response scenario, the study validates the principle of time value priority emphasized by Nisingizwe et al. (35) and provides a theoretical fulcrum for establishing a multidimensional evaluation system for medical UAVs.

For medical UAV delivery projects that have entered the trial operation stage, the focus should be shifted to improving the performance of the aircraft, increasing the number of aircraft, optimizing the efficacy of the on-board equipment, ensuring professional staffing, and strengthening the routine maintenance of the aircraft, i.e., focusing on the enhancement of Factors F_9_, F_14_, F_15_, F_16_, and F_18_ (since the demand for medical supplies is an objective factor in the present study, F_18_ was not included in the optimization). Policy support (F14) corroborates Aggarwal’s (23) assertion on the specialized adaptation of medical drones, which shares both commonalities with traditional logistics research (28) that emphasizes the principle of infrastructure prioritization and essential differences due to the biosecurity nature of medical transport. Such findings provide a footnote to the specificity of the medical scenario to the theory of dynamic adaptation of regulatory frameworks proposed by Röper et al. (39). Although these construction measures fall under the category of infrastructure by nature, they have a significant positive impact on the willingness of medical personnel to use drones for medical material delivery. The previous analyses indicate that hospital doctors who have been involved in experimental or pilot activities of drone delivery are overall more inclined to accept this mode of delivery as compared to those who have no relevant exposure experience. Therefore, strengthening such infrastructure work will not only help to expand the coverage and task frequency of delivery operations in the future but also significantly enhance the trust and support of medical personnel for drone delivery of medical materials, thereby promoting the promotion and normalized application of this technology.

Based on previous research, the implementation of medical drone technology in urban settings must prioritize the acceptance levels among both the general public and healthcare professionals (73). Currently, globally, no hospitals or organizations have been able to achieve large-scale, regular use of drones for the delivery of medical supplies. This is mainly attributed to the late start of the field internationally, and the construction related to high drivers is still in progress. Combined with the model analysis in this paper, it is recommended that after better completion of the first two construction phases, the focus should be on optimizing the following aspects: improving the efficiency of drone deployment (F_2_), lowering the cost of distribution (F_3_), strengthening the safety of distribution (F_4_), improving the level of operation and management (F_13_), and enhancing the ability of emergency response (F_8_). These optimization measures have a significant contributing effect on increasing the willingness of medical personnel to use drones to deliver medical supplies in the future. Especially in scenarios where drones deliver high-value or scarce medical supplies, how to effectively address the above key issues will play a crucial role in promoting the widespread application and normalized operation of drone delivery technology.

## 7 Conclusion

The main obstacle to encouraging the normalized use of the technology is improving the medical staff’s acceptance of UAV medical delivery. This study’s ISM hierarchical analysis reveals that the fundamental environment for technology promotion is made up of bottom-level drivers like legal regulation and policy support, middle-level dependency factors like equipment performance and maintenance systems that have a direct impact on how well technology is implemented, and top-level high-dependency factors like cost control and delivery efficiency that determine whether normalized application is feasible. Compared with existing studies focusing on UAV hardware improvement and path optimization, this paper reveals the non-physical barriers hindering the implementation of the technology from the user’s perspective and provides differentiated strategies for different construction phases: in the early stage of the construction phase, it is necessary to build a good development environment; in the trial period, the infrastructure should be upgraded; and in the promotion phase, it is necessary to optimize the deployment efficiency, control the distribution cost, strengthen the safety performance, and improve the operation and management capability.

It should be noted that although this study has clarified the structural relationship of the key influencing factors through hierarchical analysis, it has not yet quantified the specific intensity of the effect of each factor on willingness to use, which requires the development of a special assessment tool in subsequent studies. Second, because the current activities of drone delivery of medical supplies are in their infancy all over the world, some of the findings need to be validated in long-term follow-up studies. Finally, this paper only analyzes from a macro perspective, and in future practical work, it is necessary to strengthen the micro part of the research, which can more efficiently contribute to the gradual promotion of this activity.

## Data Availability

The raw data supporting the conclusions of this article will be made available by the authors, without undue reservation.
